# How do parents deal with their children’s chronic kidney disease? A qualitative study for identifying factors related to parent’s adaptation

**DOI:** 10.1186/s12882-020-02170-4

**Published:** 2020-11-25

**Authors:** Fatemeh Khorsandi, Naser Parizad, Aram Feizi, Masumeh Hemmati MaslakPak

**Affiliations:** 1 Department of Medical - Surgical Nursing, School of Nursing and Midwifery, Urmia University of Medical Sciences, Urmia, IR Iran; 2 Patient Safety Research Center, Urmia University of Medical Science, Urmia, IR Iran; 3 Department of Management, Psychology, Community, and Fundamental nursing, Urmia, IR Iran; 4 Center for Mother and Child Obesity Research Center, Urmia University of Medical Sciences, Urmia, IR Iran; 5 Nursing and Midwifery Faculty, Campus Nazlu, 11 KM Road Seru, Urmia, West Azerbaijan 575611-5111 Iran

**Keywords:** Adaptation, Parents, Children, Chronic kidney diseases, Qualitative study, Iran

## Abstract

**Background:**

Parents’ adaptation affects the health outcomes of children with chronic kidney diseases (CKD). Identifying factors that affect parents’ adaptation is necessary to understand their adaptation status. This study aims to explore factors related to the adaptation of parents who have children with CKD.

**Methods:**

This was a qualitative study with a content analysis approach. Seventeen parents of children with CKD were selected by using purposive sampling. The leading researcher performed semi-structured, in-depth, face-to-face interviews to collect data. Conventional content analysis was used to analyze data.

**Results:**

Two main categories extracted from the data were “adaptation facilitators” and “adaptation barriers.” Adaptation facilitators were supported by three sub-categories: “social support”, “family capability” and “spiritual beliefs”. Four sub-categories of “adaptation barriers” were revealed as: “family-related barrier*s*,” “mental stress by others,” “the chronic nature of the disease,” and “unfavorable treatment conditions.”

**Conclusions:**

Identifying the factors influencing parental adaptation helps the medical staff to make the necessary interventions to support the parents. According to this study, increasing parent access to the required information, supporting them financially and emotionally, and helping them identify support resources can facilitate their adaptation to their child’s chronic illness. Also, identifying and eliminating adaptation barriers can help parents deal better with their child’s chronic disease.

**Supplementary Information:**

The online version contains supplementary material available at 10.1186/s12882-020-02170-4.

## Background

The number of children suffering from chronic diseases has increased in recent decades. It is predicted that the increasing trend will continue due to genetic and social-behavioral changes [1]. Chronic kidney disease (CKD), with a prevalence of 18 patients in one million people, is an irreversible and often progressive disease related to permanent damage to the kidney [2]. CKD threatens the lives of affected children with complications such as infections, bone diseases, low growth, and renal failure [3]. Chronic disease affects not only the child, but the family [4] including all aspects of parents’ lives, such as their occupation, psychological and social health, physical and emotional health [5], and family and social relations [6]. Parents of children with CKD play a crucial role in managing their child’s disease [7]. In addition to the roles of ordinary parents, these parents substitute as care coordinators, medical experts, systems advocates, and their children’s representatives [8]. Having children with chronic diseases may lead to more significant responsibilities than ordinary parents [9].

These parents suffer from various psycho-social related stress [10]. Most of the time, they are exhausted, depressed, anxious, have a low-quality of life, and are worried about their child’s future. Such intense pressure may lead to adverse effects on the child’s health outcomes and their medical treatment, as well as being debilitating for the parents [11]. Thus under such circumstances, parents find it challenging to balance their unwell child’s needs with their other responsibilities [12]. Therefore, such parents are sometimes called hidden patients [13]. These parents apply adaptation strategies to reduce tension and anxiety and adapt to their new conditions to react to the family’s stressful situations, deal with difficult situations, and maintain the whole family’s performance [14, 15].

Parents’ adaptation indicates a process by which a family manages the needs related to disease control based on available resources. Parents who are poorly adapted to their child’s illness may be involved in their children’s distress [8]. Due to the lack of adaptation, the child and their family are exposed to upcoming unpleasant consequences. These consequences may include mental health problems, low family quality of life, marital conflict, and negative effects on the child and their siblings [16]. Various factors are involved in the parents’ adaptation to their child’s chronic illness [17]. These factors must be identified in order to understand parents’ adaptation status [18]. Considering the influence of parents’ adaptation on the overall health outcome of children with chronic diseases [19], the medical team must help the family adapt to the new care situations and emotional problems associated with it [18]. However, this support is not fully implemented for these families [17].

Moreover, research regarding caregivers of chronic patients’ adaptation is limited, and the focus is more on quantitative studies than the experiences of caregivers [20]. Qualitative research is the appropriate method to access individuals’ lived experience and their inner world. A qualitative study can also help clarify the ambiguous and unknown phenomenon [21]. Since the parents’ adaptation and related factors are a vague and unknown phenomenon, this study was conducted to explore factors related to the adaptation of parents who have children with CKD.

## Methods

### Study design and setting

We used a qualitative study with a content analysis approach to explore parent experience and gain a more profound understanding of the factors that influence their adaptation. This study was conducted in Shahid Motahari pediatric hospital of Urmia in Iran.

### Participants

Participants were parents of children with CKD who were admitted to the nephrology department. We used purposive sampling to recruit the participants. First, we referred to the nephrology department and selected parents who were eligible to enter the study. Then, we conducted an informal face-to-face conversation to assess the parent’s ability to express experiences. After viewing the parents’ ability to express experiences, we explained the study’s purpose and process, their role in the study, and the confidentiality of the information. We also notified them that they could leave the study at any time. Then, we obtained verbal and written consent and asked permission to record the interview. Next, we invited parents to the study and determined the time and place of interview based on their convenience. We conducted all the interviews in a private, familiar and quiet room in the nephrology department after visiting hours. In those hours, doctors’ visits and nursing care were completed, and family visiting hours were usually finished. We invited 17 parents to participate in the study. None of the invited parents declined our invitation. Thus, 17 parents of 15 children with CKD were recruited to participate in the study. Inclusion criteria were the willingness to participate in the study, the ability of parents to express experiences, not being a single parent, more than six months passed since the child*’s* diagnosis and finally, parents not having a mental illness.

### Data collection

The leading researcher performed data collection by using interviews and field notes from September 2018 to September 2019. She had passed six credit hours of qualitative research methodology before performing the interviews. She worked in the Nephrology unit in the pediatric hospital. She was interested in the study topic and performed the interviews after obtaining permission from the parents. Semi-structured, in-depth, and face-to-face interviews were conducted. Before starting the formal interview, she briefly spoke with the participant about everyday issues to create a friendly atmosphere and a sense of comfort for the participant to express their experiences. After participants completed the demographic questionnaires, the interview began with a general and open-ended question, followed by probing questions based on the parents’ responses. She used a previous similar study to guide the first interviews [22]. She began with general open-ended questions, including “How did you deal with your child’s illness?” Based on the participants’ answers, probing questions were asked to obtain a complete understanding of their experiences. The probing questions included “What do you mean by that?” or “would you please explain more about that?” There was no need to repeat any of the interviews. The interviews lasted for 20 to 100 min, with an average of 37.76 min. Each interview was digitally recorded, transcribed, and analyzed after it is conducted. The process continued until data saturation was achieved, which indicated that no new additional concepts were identified [23].

### Data analysis

Data were analyzed using MAXQDA10 software. A conventional six-step content analysis [24] was used to analyze data and including:

1. reaching a general understanding by immersion and reading the transcription several times to detect primary ideas
2. creating primary codes in the transcription by re-reading line by line
3. searching for and determining categories and sub-categories
4. reviewing and discovering the relationship between categories and sub-categories
5. labeling and describing the categories and sub-categories
6. interpreting and presenting the final report of the analysis.

The leading author performed the data analysis, and the other authors participated and monitored the coding process. The research team had several meetings and discussions before reaching an agreement on the coding and categorization. We used the COREQ checklist to improve quality reporting in this study (see supplementary files).

### Rigor

Guba and Lincoln’s criteria were used to ensure the precision and accuracy of the data [25]. The data’s credibility increased through prolonged engagement with the data and reviewing data with experts and the participants. Conformability was achieved by careful recording and precise reporting of the research process and findings. It was also attempted to prevent the researcher’s bias during data collection and data analysis. The entire study was recorded carefully with step by step repetition and audit performed to confirm dependability. To obtain transferability, external reviewers precisely reviewed and confirmed the data.

## Results

### Demographic characteristics of the participants

Of all 17 parents who participated in the study, 11 were mothers, and 6 were fathers. Seven parents had one child, nine had two children, and one had three children. The duration of childhood illness was between 7 and 144 months (60.47 ± 44.03). Four parents had primary education, three parents had a middle school education, seven parents had a high school diploma, and three had a bachelor’s degree. Of the children’s diseases, nine cases were nephrotic syndrome; two were chronic renal failure, one was secondary to Familial Mediterranean fever (FMF), one case was secondary to Cystinosis, one was severe fetal hydronephrosis secondary to kidney stones, and one was neurogenic bladder (Table 1).

**Table 1:** Demographic characteristics of participants

No.	Gender	Number of Children	Children’s illness duration	Parents Education level	Interview duration	Job status	Children Diagnosis	Children Treatment
1	Female	2	5 years	High school Diploma	37	Housewife	Renal failure secondary to FMF	Conservative treatment
2	Female	2	2 years	Primary education	60	Unemployed	Severe fetal hydronephrosis secondary to kidney stones	Surgery & conservative treatment
3	Male	2	7 years	Middle school	41	Self-employment	Nephrotic syndrome	Conservative treatment
4	Female	1	4 years	Primary education	24	Housewife	Neurogenic bladder	Clean intermittent catheterization (CIC)
5	Male	2	5 years	High school Diploma	30	Self-employment	Husband of Participants 1	
6	Female	1	12 years	High school Diploma	33	Self-employment	Nephrotic syndrome	conservative treatment
7	Male	1	11 years	Bachelor’s degree	60	Employee	Chronic Renal Failure	Peritoneal Dialysis, transplanted
8	Male	2	7 years	High school Diploma	20	Self-employment	Nephrotic syndrome	Peritoneal Dialysis
9	Female	1	2 years	Primary education	24	housewife	Chronic Renal Failure	Peritoneal Dialysis
10	Female	2	2 years	High school Diploma	40	Housewife	Nephrotic syndrome	Conservative treatment
11	Female	2	3 years	Bachelor’s degree	25	Housewife	Nephrotic syndrome	Conservative treatment
12	Male	2	2 years	High school Diploma	41	Unemployed	Husband of Participants 10	
13	Male	1	7 months	Middle school	35	Worker	Renal failure secondary to Cystinosis	Conservative treatment
14	Female	2	2 years	Middle school	20	Housewife	Nephrotic syndrome	Conservative treatment
15	Female	3	10 years	Primary education	100	Housewife	Nephrotic syndrome	Conservative treatment
16	Female	1	3 years	Bachelor’s degree	30	Self-employment	Nephrotic syndrome	Conservative treatment
17	Female	1	4 years	High school Diploma	35	Housewife	Nephrotic syndrome	Conservative treatment

### Categories

**Fig. 1:**
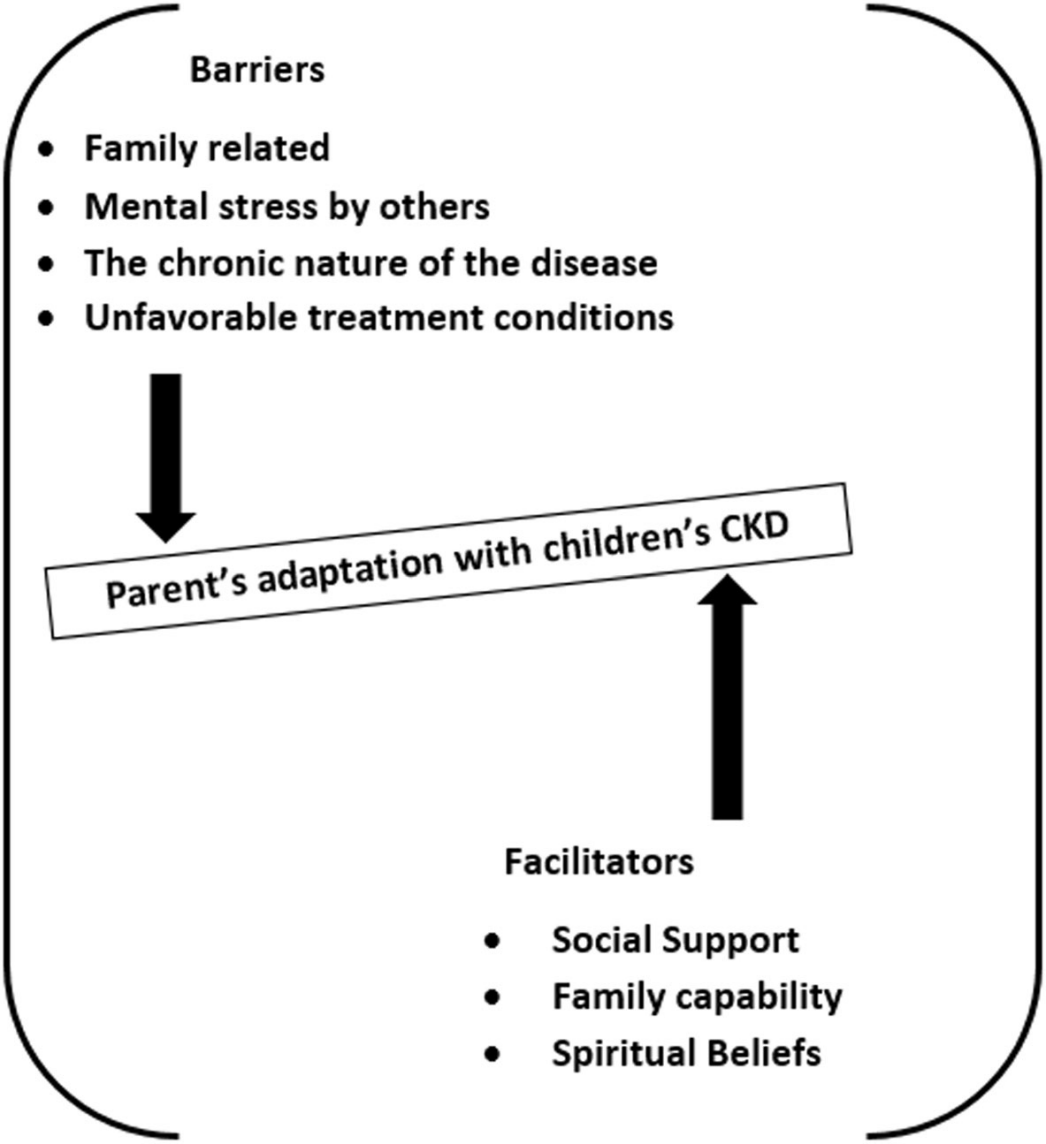
Schematic model of factors influencing parents’ adaptation

“Adaptation facilitators” and “adaptation barriers” emerged as two overarching categories from the data. Adaptation facilitator, followed by three sub-categories of “social support,” “family capability,” and “spiritual beliefs.” Adaptation barriers were supported by the following four sub-categories: “family-related barriers,” “mental stress by others,” “the chronic nature of the disease” and “unfavorable treatment conditions.” (Tables 2 and 3). (See Fig. 1).

**Table 2:** Categories, sub-categories, and primeary concepts of the study

Categories	Sub-categories	Primary concepts
Adaptation facilitators	Social support	* Hospital personnel assistance * Family Support * School personnel assistance * Support of colleagues
	Family capability	* Parents financial ability * Child’s educational success * Good relationships between parents * Parents similar past experiences
	Spiritual beliefs	* Appealing to God * Divine wisdom
Adaptation barriers	Family-related barriers	* Lack of parents’ information about illness * Parents’ concern about uncertain future * Families’ financial concerns * Restrictions in family recreation * Restrictions on family relationships * Children’s problems
	Mental stress by others	* Pity from acquaintances * Blaming parents by acquaintances * Peer negative reactions
	The chronic nature of the disease	* Frequent disease recurrence * Frequent hospitalizations * Need for constant care * Invasive treatment and diagnostic procedures
	Unfavorable treatment conditions	* Lack of medical facilities * High treatment costs

**Table 3:** Categories, subcategories, primary concepts and quotations of the study

Categories	Subcategories	Primary concepts	Quotation
Adaptation facilitators	Social support	* Hospital personnel assistance	“Nurses here are so sweet. They explain everything to me. You have to wash your and your sons’ hands more often. Whenever you come to the hall, you have to wear a mask. Your son should not go out at all in the fall and winter” [p15]. “… The nurses here give us a lot of consolation. Whenever I cried, they came in and talked to me about the other patients’ situations and gave me encouragement” [p9].
		* Family Support	“If I need help, I always ask my mom to come and help me. For example, she comes and helps me with household chores” [p15]. “… My husband is always supportive. He helps me by taking care of the kids. He takes them to the doctor, brings them back …” [p10].
		* School personnel assistance	“When my son is in the hospital, his teacher comes by to teach him” [p5]. “… Because of his disease, he is absent everyday … even his principle comes home to teach him” [p7].
		* Support of colleagues	“Fortunately, my boss is very cooperative. Once, when my son was hospitalized in Tehran, he agreed to give me off, and I was out of work for 40 days” [p7]. “… At work also, the burden is on my colleague. Sometimes, I am out for one or two days. My colleagues help me out and cover for me …” [p8].
	Family capability	* Parents financial ability	“I always thank God, we don’t have a financial problem. I can not imagine what we should do if we don’t have money to take care of our sick child!!! May God help those who do not effort medications and treatment costs” [p1]. “If you have a sick kid and also you are broke, it would be a disaster and unbearable. But, luckily, I didn’t have any financial problem in the past 11 years …” [p7].
		* Child’s educational success	“Although he is sometimes hospitalized and cannot go to school, his grades are good, and teachers are happy with him” [p4]. “He is a good student. I take him to school once a month. He takes his exams. Teachers say his grades are excellent” [p11].
		* Good relationships between parents	“My husband is like a pillar for our family. He always pays attention to me, he doesn’t let me down” [p10]. “… Despite all these difficulties, we (my husband and me) love each other. So, we’ve gone through all these problems …” [p4].
		* Parents similar past experiences	“My first kid has the same problem. I got used to the situation. I don’t take him to the doctor’s office for little things. I know what I have to do when my kids get sick” [p2]. My father was also on dialysis. I already had the experience of caring for a patient with kidney problems. It’s hard, but you get used to it” [p9].
	Spiritual beliefs	* Appealing to God	“My wife and I always pray to God in a hard time. Whenever we do that, we get energy” [p12]. “He (My husband) always told me to keep your faith up and let God take over our problems during hard times … Sometimes, he had huge problems in his life, but he believed that he’s backed by God. So his problems will go away and this has always been happening” [p1].
		* Divine wisdom	“This disease is from God, not human beings. Treatment is also on his hands and doctors are as tools in his hand. What can we do?” [p11]. “I say this is a divine test. I always say: God tests his servants with difficulties …” [p10].
Adaptation barriers	Family-related barriers	* Lack of parents’ information about illness	“We don’t know what all these medications are for. Where should we buy them? How should we give?” [p13]. “I don’t know anything about peritoneal dialysis … Creatinine increased up to 6. The doctor said he was on a razor edge, he should go for peritoneal dialysis. The only thing I knew about dialysis was this catheter that they enter from here (pointing to the neck); I said if my son had that (catheter), I couldn’t live anymore” [p7].
		* Parents’ concern about uncertain future	“When will this kid be well? How long should he take these medications? Is he going to get better? ...” [p10]. “I really worry about his future all the time. I always ask (myself) whether he will get well when he grows up. Parents don’t stay with them (children) forever …” [p11].
		* Families’ financial concerns	“My son is hospitalized more often, and we have to leave home and bring him here. When he is in the hospital, my husband stays in the hospital yard all night because we can’t effort to pay for the hotel” [p1]. “Sometimes, I have to borrow from my family to get her medications. Things are getting really tough” [16].
		* Restrictions in family recreation	“I don’t let my other child go to the park or play somewhere. Because whenever he goes out to play with his friends. He asks me why I don’t go out to play. That’s why they both stay at home” [p11]. “Everybody likes to go and enjoy parties or trips, but … these children take corticosteroids, their body is sensitive … so we can’t go out much …” [p10].
		* Restrictions on family relationships	“My son is 8 years old and he is still on a diaper. So, I try to limit the family visits” [p7]. “I don’t go anywhere because of my kid. I live in my mother-in-laws house. They usually go out and attend ceremonies … But, I stay home to take care of my kid” [p11].
		* Children’s problems	“Ever since my daughter has been using prednisolone. She gets nervous when the house gets crowded, or there is a lot of noise around her” [p6]. “His teacher told me that your son is a little depressed. I asked him what is wrong. He said, leave me alone. Everyone eats puffy cheese and chips, but I can’t … “[p15]. “My son doesn’t go to school. He went to school once; he had eaten ice cream with his friends. Then, he was hospitalized for 1 year … so we hired a teacher to teach him at home …” [p11].
	Mental stress by others	* Pity from acquaintances	“Every time my co-worker talks about my daughter with pity, I get agitated” [p6]. “Some people say: “oh, this (child) is sick; don’t tell him anything!” … It really pisses me off, and I tell them, “what’s wrong with him? Every kid gets sick...” [p10].
		* Blaming parents by acquaintances	“My friends blame me that my daughter would not get sick if I did not marry this man” [p6]. “My mother-in-law always says it is our fault (my husband and me) that he’s sick. Crying is the only thing I do and ask what I should do?” [p9].
		* Peer negative reactions	“One day, my son came home crying. I said, “Darling, why are you crying?” He told me that his friends did not let him play. They said he can’t play because he has cancer” [p5]. “… Taking corticosteroids made her (my daughter) obese... Sometimes, her friends make fun of her, it really bothers me …” [p8]
	The chronic nature of the disease	* Frequent disease recurrence	“This is the third time my son is taking this medication ... he had stopped two months ago. He has to start again because of its recurrence” [p12]. “… She takes corticosteroids every day. Her disease gets better for a while. Later, when we try to decrease and stop her medications gradually, again, her disease gets worse …” [p8].
		* Frequent hospitalizations	“My son has been hospitalized about 20 times so far. We both are sick and tired of being in the hospital” [p10]. “It’s been passed seven years since my daughter’s illness. During this time, I had to hospitalize her again and again” [p3].
		* Need for constant care	“When my son discharged home. The nurse taught my wife how to do Peritoneal Dialysis. We have to dialyze him regularly. We should pay attention to the fluid coming back from dialysis. I feel so stressed out sometimes” [p7]. “His school is far from home. I have a hard time. When he is at school, I have to go to school every 3 h and insert his catheter …” [p4].
		* Invasive treatment and diagnostic procedures	“I always thought my daughter would be treated with medications ... but when I found out, they had to take her to the operating room and put a catheter in her abdomen (peritoneal dialysis catheter) … I had a hard time breathing [p8]. “The doctor told me, “Your kid needs to have a biopsy.” I said, “What does that mean?“He said that they should take a sample from his kidney. When I heard that my world came apart” [p15]
	Unfavorable treatment conditions	* Lack of medical facilities	“Doctor told me your son needs a color biopsy, and we don’t have it here. You must go to Tehran. I was so depressed” [p5]. “The boy (transplantation donor) had come from Tehran for pre-surgical anesthesia assessment. So that, he supposed to get a consultation on Tuesday, hospitalize on Thursday, and go under the surgery on Saturday. He (laboratory director) said, unfortunately, the solution (necessary for tissue typing test) was not available. I asked what that is. He said the (required) material for this test came from Germany. It is not available now. [p7].
		* High treatment costs	“If it was just the medications costs, we wouldn’t have any problem. I took my son to a nutritionist and cost me too much money” [p13]. “During these years, sometimes my son had been hospitalized for a month. Well, it cost us too much money, and we had a hard time to survive” [p10].

### Adaptation facilitators

#### Social support

The participants emphasized this item as one of the significant factors that help them adapt to hard situations. The following primary concepts emerged from this sub-category: “hospital personnel assistance,” “family support,” “school personnel assistance,” and “support of colleagues.”

#### Hospital personnel assistance

Some parents talked about how the guidance and education they received from the nurses, doctors and resources increased their knowledge. This education, in turn, enabled them to provide high-quality care for their child with a more peaceful mind. Some of the participants stated:

“… The nurses here give us a lot of consolation. Whenever I cried, they came in and talked to me about the other patients’ situations and gave me encouragement” [p9].

“At the time of discharge, the doctor explained everything so nicely. He gave me the instruction, how to give his (my son) medications, how long I should give his medications, and when he should stop taking them …” [p1].

#### Family support

Parents voiced that caring for their children with the chronic disease made them feel exhausted. In such circumstances, their family’s support made it easier for them to go through. This support was either emotional or financial. Some of the participants’ experience was as follows:

“… His treatment costs 180 million Rials the last time he was in the hospital. Can you believe that? I didn’t even have one Rial. My brothers paid it off.” [p7].

“… My husband is always supportive. He helps me by taking care of the kids. He takes them to the doctor, brings them back …” [p10].

#### School personnel assistance

Some of the parents discussed how teachers or principals helped their children during their acute illness. Teachers went to their homes to teach them so that they would not miss their lessons. One of the parents said:

“… Because of his disease, he is absent everyday … even his principle comes to our home to teach him” [p7].

#### Support of colleagues

Some parents mentioned their colleagues’ assistants and how supportive they were whenever facing difficult situations. One of the parents stated:

“… At work also, the burden is on my colleague. Sometimes, I am out for one or two days. My colleagues help me out and cover for me …” [p8].

#### Family capability

Family capability is one of the adaptation facilitators that parents repeatedly talked about. The primary concepts related to this sub-category include: “parents’ financial ability,” “child’s educational success,” “good relationships between parents,” and “parents’ similar past experiences.”

#### Parents’ financial ability

Participants mentioned that some factors, such as their financial ability, made it easier for them to tolerate their child’s disease. A father voiced:

“If you have a sick child and you are broke, it would be a disaster and unbearable. But, luckily, I didn’t have any financial problems in the past 11 years …” [p7].

#### Child’s educational success

Another comforting factor for parents was a child’s good education status. Participant 11 stated:

“He is a good student. I take him to school once a month. He takes his exams. Teachers say his grades are excellent.*”* [p11].

#### Good relationships between parents

According to the participants, another supportive factor for parents was their happy and peaceful relationship during this overwhelming and challenging situation. One parent stated:

“… Despite all these difficulties, we (my husband and I*)* love each other. So, we’ve gone through all these problems …” [p4].

#### Parents similar past experiences

Analyzing parents’ experiences indicate that the ups and downs of life have improved parents’ tolerance and made them more capable of managing their child’s disease.

“My father was also on dialysis. I already had the experience of caring for a patient with kidney problems. It’s hard, but you get used to it” [p9].

#### Spiritual beliefs

Spiritual beliefs were another facilitating factor that effectively helped parents get along with their child’s disease. Parents believed “appealing to God” and “divine wisdom” helped them to tolerate the hard situation.

#### Appealing to god

Participants discussed that faith and belief in God have brought them tranquility and increased their ability to handle their problems. A mother stated:

“He (My husband) always told me to keep your faith up and let God take over our problems during hard times … Sometimes, he had huge problems in his life, but he believed that God backed him. So his problems will go away, and this has always been happening” [p1].

#### Divine wisdom

Participants mentioned that life’s difficulties have been for a divine reason and described their child’s disease as God’s test. Parents shared their experience as follows:

“I say this is a divine test. I always say: God tests his servants with difficulties …” [p10].

“This disease is from God, not human beings. Treatment is also on his hands, and doctors are tools in his hand. What can we do?” [p11].

### Adaptation barriers

#### Family-related barriers

This sub-category contained the primary concepts of “lack of parents’ information about the illness,” “parents’ concern about the uncertain future,” “families’ financial concerns,” “restriction in family recreation,” “restrictions on family relationships” and “children’s problems.”

#### Lack of parents’ information about the illness

Based on statements by the participants, the lack of necessary information about their child’s disease, and how to care for their sick child made them confused. One participant voiced:

“I don’t know anything about peritoneal dialysis … Creatinine increased up to 6. The doctor said he was on a razor’s edge; he should go for peritoneal dialysis. The only thing I knew about dialysis was this catheter that they enter from here (pointing to the neck); I said if my son had that (catheter), I couldn’t live anymore.” [p7].

#### Parents’ concern about the uncertain future

According to the participants, the uncertain nature of the disease and the uncertainty of the child’s prognosis made the parents feel concerned about their child’s future. A mother voiced her concern as follows:

“I really worry about his future all the time. I always ask (myself) whether he will get well when he grows up. Parents don’t stay with them (children) forever …” [p11].

#### Families’ financial concerns

Families’ financial problems were dominant in the parents’ interviews. Most parents complained about financial difficulties. Participant 4 voiced:

“My son is hospitalized more often, and we have to leave home and bring him here. When he is in the hospital, my husband stays in the hospital yard all night because we can’t afford to pay for the hotel.” [p1].

#### Restrictions in family recreation

The majority of the participants explained that they restricted their recreational activities and family trips due to their child’s limitations for attending social events due to weakness of their immune system. One parent shared her experience as:

“Everybody likes to go and enjoy parties or trips, but … these children take corticosteroids, and their body is sensitive … so we can’t go out much …” [p10].

#### Restrictions on family relationships

Participants expressed that they had to limit their social interactions due to their child’s special diet and disease complications.

“I don’t go anywhere because of my kid. I live in my mother-in-law’s house. They usually go out and attend ceremonies … But, I stay home to take care of my kid.” [p11].

#### Children’s problem

Participants described that the disease, its complications, and the medications’ side-effects limit children in their normal functions and daily activities, thus disrupting their social growth.

“My son doesn’t go to school. He went to school once; he had eaten ice cream with his friends. Then, he was hospitalized for one year … so we hired a teacher to teach him at home …” [p11].

According to the parents, the feeling of being special and different from other children, hearing hallucinations, yearning, being bored, fear of loneliness, depression, and irritability are among the mental health problems. Moreover, repeated infections, rapid fatigue, and anemia are among the physical health problems that these children suffer from. Parents described:

“… My daughter suffers from too much hair all over her body. She’s gained weight, and so she feels socially awkward …” [p8].

“… They constantly inserted a catheter for my child. She couldn’t pee herself, so she repeatedly got urinary tract infections *…”* [p16].

#### Mental stress by others

The parents discussed pressure from acquaintances. They voiced that it is hard to withstand their pity and blame. The inappropriate behaviors of their child’s peers made it even more difficult to handle the situation.

#### Pity from acquaintances

Most parents experienced people talking about their sick children. They believed that it is terrible since they feel weak, incapable, and humiliated. A young mother described her experience as:

“Some people say: “oh, this (child) is sick; don’t tell him anything!” … It really pisses me off, and I tell them, “What’s wrong with him? Every kid gets sick...” [p10].

Participant 4 shared her experience as follows:

“Some people see my son and say, “oh, poor child.” It makes me feel really sad …” [p4].

#### Blaming parents by acquaintances

Parents talked about how others assigning blame made them feel guilty about their child’s illness. This feeling, in turn, leads to psychological distress in parents. One of the mothers said:

“My mother-in-law always says it is our fault (my husband and me) that he’s sick. Crying is the only thing I do and ask what I should do?” [p9].

#### Peer negative reactions

According to parents’ responses, their child had a body change resulting from a combination of medication side effects. So, their child peer made fun of them and hurt their feelings. A father stated:

“… Taking corticosteroids made her (my daughter) obese... Sometimes, her friends make fun of her, and it really bothers me …” [p8].

#### The chronic nature of the disease

The chronic nature of the disease was one of the inhibiting factors with the following primary concepts: “frequent disease recurrence,” “frequent hospitalization,” “need for constant care,” and “invasive treatment and diagnostic procedures.”

#### Frequent disease recurrence

Frequent recurrence of the disease and restarting medications at higher doses was another concern of the parents. One of the parents said:

“… She takes corticosteroids every day. Her disease gets better for a while. Later, when we try to decrease and stop her medications gradually, again, her disease gets worse …” [p8].

Another parent described:

“It had been a little more than a year since she was in the hospital. I don’t know if she caught a cold or what! Her eyes got swollen again. We took her to the doctor’s office, and we are here now.” [p14].

#### Frequent hospitalization

The child’s frequent hospitalizations was another point that parents talked out. One parent stated:

“It’s been seven years since my daughter’s illness. During this time, I had to hospitalize her again and again.” [p3].

#### Need for constant care

Some participants complained about feeling exhausted caused by the necessity of constant care for their children. They believed their entire lives were affected by their child’s illness. One of the participants shared her experience as follows:

“His school is far from home. I have a hard time. When he is at school, I have to go to school every 3 hours and insert his catheter …” [p4].

#### Invasive treatment and diagnostic procedures

Most parents believe that the procedures performed on children were harsh to bear. Parents talked in this regards as follows:

“I always thought my daughter would be treated with medications ... but when I found out, they had to take her to the operating room and put a catheter in her abdomen (peritoneal dialysis catheter) … I had a hard time breathing.” [p8].

“The doctor told me, “Your son needs to have a biopsy.“ I said, “What does that mean? “He said that they should take a sample from his kidney when I heard that my world came apart.” [p15].

#### Unfavorable treatment conditions

The participants mostly complained about improper treatment conditions. This sub-category emerged from the following primary concepts: “lack of medical facilities” and “high treatment costs.”

#### Lack of medical facilities

Participants discussed how medication shortage, lack of medical facilities, and the imported laboratory test kits delayed a child’s treatment process and distressed them. One of the parents described:

“The boy (transplantation donor) had come from Tehran for pre-surgical anesthesia assessment. He was supposed to get a consultation on Tuesday, be hospitalized on Thursday, and undergo the surgery on Saturday. He (laboratory director) said, unfortunately, the solution (necessary for tissue typing test) was not available. I asked what that is. He said the (required) material for this test came from Germany. It is not available now.” [p7].

One of the fathers shared his experience as follows:

“… The doctor prescribed some medications for my son. I can’t find them anywhere, and I had to go to Turkey to get them. It is unbelievable.” [p13].

#### High treatment costs

Analyzing the data showed high *cost* of medications, doctor visits, and frequent hospitalizations impose a huge financial burden on the parents. Participant 10 stated:

“During these years, sometimes my son had been hospitalized for a month. Well, it cost us too much money, and we had a hard time to survive.” [p10].

## Discussions

Adaptation is a dynamic process that varies depending on the individuals, situations, and requirements [26]. After analyzing the data, various factors were identified as adaptation barriers and facilitators in parents whose children suffer from CKD. First, we will discuss the factors that facilitate parents’ adaptation to their child’s chronic disease. *Social support* was one of the facilitators in adapting the parents of children with CKD. Provision of information and emotional support from medical staff, financial and emotional support of family, teachers’ help in preventing the child from falling behind during hospitalization, the cooperation of officials and colleagues in the workplace were among the factors that had a positive effect on parental adaptation. Similar to our findings, the previous studies showed that parents who had social support could better adapt to their child’s cancer [27–29]. A literature review confirmed that parents who had children with CKD use coping strategies such as social, emotional, and financial support and counseling services from family, friends, school, and medical teams [10, 11, 30, 31].

The participants talked about *family capability* as a facilitating factor in this study. Parents who have good financial ability, their child is successful in the school, have a good marital relationship, and have similar experiences could adapt to their children’s chronic illness more efficiently. They voiced that the existence of family capability increases their hope and tolerance, making it easier for them to adapt to their child’s disease. Limited studies have been conducted in this regard. In line with our findings, Wiedebusch et al. reported that parents of children with renal failure used the positive aspects of life as a positive coping strategy [10]. Parents who have a high family income and financial ability make them feel empowered to continue to treat their children despite the high costs [32], a healthy parent’s relationship, emotional support of a spouse, a Child success and educational achievements encourage parents and lead them to successful adaptation [31, 33, 34]. These findings are consistent with our results. However, a study by Oskooi et al. showed that past painful experiences could have a positive or negative effect on the adaptation of parents whose children have diabetes [35]. It may depend on the personalities of the individuals. Some can tolerate high-level crises without experiencing physical and mental problems [36].

*Spiritual beliefs* were among the facilitating factors indicated by most parents. The parents rescue themselves from anxiety and depression caused by their child’s disease through their spiritual beliefs. Similar to our findings, recent studies showed that most parents of children with cancer use spiritual beliefs to adapt emotionally to stressful situations, and they had a better adaptation compared to non-religious ones [29, 37]. Similarly, parents of children with CKD use coping strategies such as religious beliefs to adapt to their child’s chronic disease [10]. Thus, spiritual beliefs create hope, motivation, positivity, and emotional support network for parents.

Participants discussed some factors that challenged their adaptation. *Family-related barriers* were one of the adaptation barriers in this study. Lack of essential information about their child’s illness and care and the uncertainty of the prognosis of the child’s disease made them confused and concerned about their child’s future. In agreement with our results, Pate reported that chronic diseases of children with an unknown prognosis and treatment could disrupt parents’ daily activities and threatens the entire family system [38]. In Hutchinson et al. study, uncertainties in the trajectory of cancer have been recognized as a significant obstacle to successful adaptation in the family [39]. Previous studies determined that parents need more information regarding child illness, treatment, diet, and medication to adapt successfully to the stressful situation [11, 31, 40]. Thus, medical staff, as an important source of information, can provide the information that parents need to facilitate their adoption. Children’s problems, families’ financial concerns and limitations caused by the child’s chronic disease was another annoying element for parents. They voiced the limitations of the children in social interactions due to special diets and the weakness of their immune system. They also talked about physical complications caused by illness or medications that may socially isolate these children and cause them mental problems like depression. The parents had to restrict their social interactions and family recreation, to prevent complications of the disease. There is limited research on this topic. In consonance with our results, Piran et al. showed that social isolation is more widespread among people who provide care for children with chronic diseases [41]. The Goble study revealed that fathers who take care of children with chronic diseases lose their previous social activities [42]. Children’s problems and family restrictions can hurt parents and lead to unsuccessful adaptation.

*Mental stress by others* was another inhibiting factor in parents’ adaptation. The participants mentioned that blaming them or talking about their child by others, and their children’s negative peer reactions caused tensions for parents and children. Limited studies have been conducted on this matter. Abedi et al. showed that parents of children with thalassemia experienced a broad range of social and family difficulties after being blamed, unfairly judged, and criticized by acquaintances [43]. These pressures can be a source of mental problems and result in negative adaptation in these parents. Thus, these parents need to receive more emotional and mental support.

*The chronic nature of the disease* was revealed as one of the barriers to parents’ adaptation. Parents experienced mental and physical exhaustion due to the chronic nature of the disease, frequent recurrence, frequent hospitalizations of the child, the need for constant care and the need for invasive diagnosis and treatment. The results of the Yamazaki et al. study in Japan indicated that mothers of children with leukemia had a poor quality of life due to the constant hospitalization of their children [44]. A study conducted by Hutchinson et al. showed that the burden of caring for children with a chronic condition was recognized as one of the biggest obstacles to adaptation of the family [39]. Several recent studies indicated that depression, high blood pressure, anemia, repeated infections, fatigue, and failure to thrive were among the prevalent complications of CKD in children with a negative impact on parents’ adaptation [2, 45, 46]. Child psychological problems and frequent hospitalizations were the challenges faced by parents of children with CKD and lead to poor family adaptation [30, 31]. Hence, parents need more effort to manage the situation and successfully adapt to challenges [30].

Unfavorable treatment conditions were another obstacle to successful adaptation in these parents. Based on the participants’ responses, high treatment costs, especially in low-income families, as well as the lack of medical facilities and having problems accessing medication and other medical services, made life difficult for these families. Recent studies indicated that parents of children suffering from chronic disease might face challenges such as financial burdens [6, 47] and difficulties accessing services [6]. The parents who take care of children with chronic diseases spend a lot of money, which may impose high pressure on the families [43]. Moreover, financial problems were serious challenges for the families [48], which can increase stress in the families and hurt their adaptation [35].

## Conclusions

Identifying the factors influencing parental adaptation helps the medical staff to make the necessary interventions to support the parents. According to this study, increasing parent access to the required information, supporting them financially and emotionally, and helping them identify support resources can facilitate their adaptation to their child’s chronic illness. Also, identifying and eliminating adaptation barriers can help parents deal better with their child’s chronic disease.

### Limitations

This study has some limitations. First, this was the first qualitative study conducted by the researcher. Thus, the researcher obtained guidance from an advisor and colleagues who were experts in qualitative research. Second, the number of fathers who participated in the study was lower than the mother participants. It is because mothers have a significant role in taking care of the child, and also fathers are too busy to stay with the child in the hospital. The authors suggest that intervention studies be designed and implemented based on the results of this study. If the results are confirmed, it can be used to help parents adapt to their child’s chronic illness more efficiently. The role of siblings was not investigated in this research. Thus, the authors recommend the role of siblings and its effect on parent adaption to be investigated in future studies.

## Supplementary Information

**Additional file 1.**

## Data Availability

The datasets used and analyzed during the current study are available from the corresponding authors on reasonable request.

## Abbreviations

CKD: Chronic kidney disease
FMF: Familial Mediterranean fever
